# Testing a conceptual Hierarchy of Effects model of food marketing exposure and associations with children and adolescents’ diet-related outcomes

**DOI:** 10.1017/S1368980023002616

**Published:** 2023-12-07

**Authors:** Bridget Kelly, Emma Boyland, Mimi Tatlow-Golden, Paul Christiansen

**Affiliations:** 1 Early Start, School of Health & Society, University of Wollongong, Wollongong, NSW 2522, Australia; 2 Institute of Population Health, University of Liverpool, Liverpool, UK; 3 School of Education, Childhood, Youth and Sport, The Open University, Milton Keynes, UK

**Keywords:** Child, Adolescent, Food, Drink, Marketing, Brand

## Abstract

**Objective:**

Children’s exposure to unhealthy food marketing contributes to poor diets by influencing the foods that children like, request, buy and consume. This study aimed to use confirmatory mediational analyses to test a hypothetical model of marketing effects, to better understand the mechanisms behind food marketing’s impacts on children.

**Design:**

Children responded to a cross-sectional online survey about their attitudes towards, and purchase and consumption behaviours of, ten frequently promoted food/beverage brands and their media use. Structural equation modelling tested *a priori* potential pathways for the effects of food marketing exposure on children’s diets.

**Participants:**

10–16-year-old children (*n* 400).

**Setting:**

Australia.

**Results:**

There was a significant positive correlation between children’s commercial screen media use and their attitudes towards brands (related to perceived social norms) and their brand purchasing behaviours, including their own purchases and requests to parents. The use of strategies to avoid advertising in commercial screen media reduced but did not remove the association between media use and brand purchases. Other brand exposures (on clothing, outdoor advertising, sponsorships) had a positive association with children’s perceived social norms about brands and their brand purchases and requests. Non-commercial screen media use was not associated with any brand-related outcomes.

**Conclusions:**

Commercial screen media use and other brand exposures were strongly positively associated with children’s perceptions and purchasing behaviours of frequently marketed food/beverages. Regulations to restrict children’s exposures to food marketing on-screen and through other media are required to reduce the effect of marketing exposure on children’s food purchasing behaviours.

Restricting children’s exposure to unhealthful food (including beverages) marketing is recognised at the highest levels of global public policy as a priority action for the prevention of poor diets, overweight and related non-communicable diseases^(1,2)^. In a series of reviews undertaken for the WHO to inform policy guidelines, food marketing was found to: be ubiquitous across media and settings with which children engage^(3)^; predominantly promote foods and beverages that contribute to unhealthy diets^(3)^ and be positively associated with children’s food preferences, choices, purchase requests and intake^(4)^. Much of the evidence focused on younger children, with relatively less on the effects of food marketing on adolescents^(4)^.

Some years ago, the authors conceptualised a Hierarchy of Effects model for food marketing, which proposed an ordered cascade of responses from food marketing exposure to cognitive (e.g. brand awareness), attitudinal and affective (e.g. emotional responses to brands) and behavioural outcomes (e.g. brand purchases and consumption) and ultimately leading to effects on diets, weight status and diet-related diseases^(5)^. This model has since been considered in evidence reviews to describe and justify important outcomes associated with food marketing exposure^(6)^ and used by governments to outline the need for policy reform on food marketing (e.g.^(7)^). Hypothesised Hierarchy of Effects model’s value lies in depicting a sequenced set of effects of food marketing exposure, such that evidence on the impacts of marketing on proximal cognitive and affective outcomes contributes to decisions about implementing policies that seek to modify more distal behavioural and health outcomes. Yet, despite the usefulness of this model for describing the range of, and interconnections between, marketing impacts, few studies have examined relationships between these outcomes. Three recent studies tested hypothesised associations between children’s television viewing (as a proxy for television food advertising exposure)^(8,9)^ or recall of food advertising on online videogame streaming platforms^(10)^ and their purchase and consumption behaviours of unhealthful foods. Using structural equation modelling, these studies found significant positive direct associations between children’s television viewing or their recall of online food advertising and their attitudes^(9,10)^, purchases^(8,10)^ and consumption^(8–10)^ of unhealthful foods. Further, commercial television viewing was indirectly associated with children’s BMI through food purchasing and with consumption through food purchase requests^(8)^. Associations between non-commercial television viewing and these outcomes were either non-significant or weaker^(8)^. Other cross-sectional surveys not applying such modelling techniques have similarly found a strong association between commercial television viewing and unhealthy food consumption, while the association with non-commercial television was weaker^(11)^. Further strengthening the causal reasoning, the association between commercial television viewing and unhealthy food consumption was found to only apply to children who reported watching advertising, including those who always or mostly watched live at the time of broadcast or who did not skip through advertising in recordings^(11)^.

Theory-testing of the Hierarchy of Effects model for food marketing supports understanding of the mechanisms behind food marketing’s impacts on children. The aim of this study was to use confirmatory mediational analyses to test relationships between children’s food marketing exposure and food brand-related outcomes along the Hierarchy of Effects pathways. In doing so, it sought to explore the likely mechanisms linking children’s food marketing exposure to unhealthy diets. By testing the indirect effect of marketing exposure on children’s food purchasing behaviours, through brand attitudes and emotional responses, the study also sought to explore the importance of brand-building in marketing communications and the development of brand personae. Brands are the unique name and/or symbol that identifies a product and distinguishes it from competitors^(12)^. Branding is a dominant feature of marketing, whereby the brand serves as an anchor for brand attitudes and attachment, and social norms related to brand use^(13)^. Strong, positive affective responses to brands are a precursor for consumer brand loyalty and market share^(14,15)^. As such, children’s positive attitudes and affective responses to brands were hypothesised to be an intermediary between food marketing exposure and behavioural outcomes, including brand purchase and consumption.

## Methods

A cross-sectional survey was used to test *a priori* potential pathways for the effects of food marketing exposure on children’s diets, based on the Hierarchy of Effects model outlined in the review paper by Kelly *et al*.^(5)^. Using structural equation modelling, children’s attitudes, emotions and attachments towards, preferences for, and intention to consume and request food brands were modelled against children’s reported purchase and consumption of food brands. Food marketing exposure, from screen media (television and online) and other media, was predicted to be positively associated with these food brand-related outcomes. Structural equation modelling is a multivariate statistical method that can be used to test interrelationships between a number of variables. It is a useful statistical approach for model testing, to assess the direct and indirect effects of pre-established relationships. The three stages of structural equation modelling analyses involved: (i) confirmation of the proposed theoretical model underpinning the relationship between food marketing exposure and children’s diets; (ii) assessing the overall fit of data to the model and (iii) evaluating the specific parameters of the proposed model.

### Sampling

Children aged 10–16 years were recruited through an online panel of Australian households registered with a market research agency (McNair yellowSquares) in November–December 2020. This research panel provider has a large research-only panel of adults, with members who have registered to be contacted to participate in surveys. Potential participants were contacted through their caregivers. Quotas were established to achieve an approximately equal split of participants by sex and age, and distribution across States/Territories and Capital cities/other based on population distribution. A sample size of 500 was sought to undertake the planned mediational analyses, based on a minimum sample size of 200 people plus 40–50 people per variable captured.

### Measurement instrument

#### Development process

Draft questions were based on earlier surveys on children’s food and food brand attitudes, attachments and preferences^(15–17)^ and social influences of eating behaviours^(18)^. Questions relating to children’s food intake behaviours were aligned to short questions in population dietary surveys^(19)^. Questions on food brand marketing exposures were based on surveys of screen media use^(11,20)^ and other media exposures^(21)^. Two rounds of formative interviews were undertaken to further inform questionnaire development. The first round used exploratory interviews with ten children (10–16 years), with representation across gender, age and socio-economic status groups. Interviews explored constructs related to food brand attitudes and behavioural intentions, to conceptualise how children understood and thought about food and beverage brands. This included the use of projective questioning (e.g. asking participants to imagine the brands as guests at a party and to describe what they might be like, intended to give an indication of brand personas) and seeking responses to rational statements (e.g. asking about agreement to statements of brand attachment, such as ‘[Brand] is just right for a person like me’). These questions sought to explore the ways that children perceived and discussed brands and were designed to assist children in considering their perceptions of food brands beyond their taste qualities. The second round of interviews involved cognitive testing of the draft questionnaire with eight children (10–16 years) to confirm interpretation of the questions (piloting). Parents were in attendance to provide their views on children’s food intake and screen use behaviours. A social research agency, with experience in undertaking commercial research and exploring consumer responses to brands, was contracted to undertake recruitment and fieldwork for the formative interviews. All interviews were conducted via videoconferencing, given COVID-19 social distancing requirements at the time, and took 40–55 min. Children participating in the formative interviews received a $50 voucher to thank them for their time.

#### Measures

Frequently promoted food and beverage brands were selected, for which children responded to brand-related questions. Brands were identified from Australian food marketing monitoring data^(22,23)^. As several brands were associated with multiple products, those to which children had varying responses during formative interviews were excluded. For example, Allen’s (confectionery brand) manufactures a range confectionery products and questions elicited an ‘it depends’ [on which product] response when children were asked to discuss their feelings about, preferences for and associations with the brand. The final list of ten brands included: McDonald’s and KFC (fast food restaurants); Pringles and Twisties (savoury crisps/chips); KitKat, M&Ms and Maltesers (chocolate); Coca-Cola (soft drink) and Monster Energy and Red Bull (energy drinks).

For each brand, children were asked to indicate their:

- Brand attitudes: by indicating their agreement (5-point Likert scale from ‘strongly disagree’ to ‘strongly agree’) with four statements: two on normative beliefs (Descriptive norms (‘Lots of people my age like [brand]’) and injunctive norms (‘Popular kids my age eat/drink [brand]’)) and two on brand attachments (‘[Brand] is right for me’ and ‘I think about [brand] regularly’).
- Emotional response to brands: Children were asked to select the emoji that best represented how they felt about the brand (7-point scale from ‘hate’ (confounded face) to ‘love’ (smiley face with love heart eyes)).
- Brand preferences: by indicating if they would serve the brands at a party they were hosting (Y/N) and if they would be willing to pay more for the brands compared with similar unbranded foods (buy/buy a cheaper product and keep leftover money).
- Brand purchase requests: how frequently they asked their parents to buy the brand (7-point scale from ‘never’ to ‘every day’).
- Brand purchases: how frequently they used their own money to buy the brand (7-point scale from ‘never’ to ‘every day’).
- Brand consumption: how frequently they consumed the brand (7-point scale from ‘never’ to ‘every day’).

Children were then asked about their media use behaviours, on which to base estimates of potential food marketing exposure. Given the earlier differential findings for commercial and non-commercial television viewing on the outcomes of interest^(8,11)^, and differences between children who actively avoided advertising and those who did not^(11)^, media use questions captured both commercial and non-commercial platforms and use of strategies to avoid marketing. Children were told to consider their media use over the last month for all visual screen-based media (television, games, social media, websites) and indicate time spent watching/playing/using media (hours and minutes) for a normal school day and a normal weekend day. A comprehensive list of all available channels/platforms was given for free-to-air, satellite and subscription television, and social and video sharing media. Gaming was listed as app, online and console games. Time spent searching and browsing websites was also recorded. Media use was asked separately for each media service/platform (e.g. Netflix, Facebook) and separately for the free version and paid version (adverting-free) of relevant services/platforms (e.g. YouTube and YouTube Premium), to allow enumeration of commercial screen media use and non-commercial screen media use. For each commercial screen media platform, children indicated if they usually watched all or most of the ads, or if they blocked half, most or all the ads by skipping over, muting or blocking. ‘Other brand exposures’ were assessed by asking if children had apps for the food brands on the mobile devices they used (for those brands with apps), or if they had seen the brand on clothing/merchandise, in sport sponsorship, in personal communications (e.g. SMS) or in outdoor advertising (all Y/N).

Demographic data were captured, with the assistance of caregivers, on children’s sex, age, suburb of residence and education level of caregivers.

### Procedure

Parents who were interested in their child potentially participating were emailed a unique link to the online questionnaire. The landing page included the participant information statement and consent for parents and, separately, information and assent for children with a check box for each to indicate consent to participate. This was followed by a set of screening questions to assess eligibility (based on age, and to achieve sex, age and geographic location quotas). The questionnaire took an average of 16 min to complete. Children’s participation was remunerated with points to the value of $3, credited to their parent/caregiver’s online panel account.

### Analyses

Data were imported into Microsoft Excel and cleaned for implausible data on media use. Weekly time spent using media (in minutes) was calculated based on the sum of individual services/platforms and multiplied by days per week (5 × weekdays + 2 × weekend days), given separately for all commercial screen media and all non-commercial screen media. The distribution in media use was large (Table 1). We retained children with media use > 3 sd from the mean, given these data are possible (very high media users with screen multi-tasking). We removed those where cumulative screen time was > 1440 min/d (i.e. > 24 h, considered implausible). Other food brand exposure was calculated as the sum of all the different ‘other’ exposures for each brand divided by the number of possible exposures (as only some included brands had apps).

**Table 1:** Sample characteristics

Variable	Mean	sd
Age	12·81	1·99
Sex (M: F)	210:190	
Commercial screen media h/week	35·08	27·76
Commercial television h/week	12·02	16·44
Commercial online h/week	23·06	22·11
Non-commercial screen media h/week	19·56	18·65
Non-commercial television h/week	14·95	15·30
Non-commercial online h/week	4·61	9·73

M:F, male: female.

All analyses were conducted using the Iavaan package in R^(24)^. As all the brand-related measures used ordinal scales and the media use measures had large positive skews, the models (confirmatory factor analyses and structural models) were fitted using maximum likelihood with robust standard errors^(25)^. Model fit was assessed using the normed Χ^2^ (Χ^2^/df) with values below three indicating a good fit, and comparative fit index (CFI) values of ≥ 0·95 being considered a good fit and > 0·90 being acceptable. The root mean square error of approximation (RMSEA) parsimony adjusted measure was also used, with values ≤ 0·06 being a good fit and ≤ 0·08 being acceptable. Finally, the standardised root mean residual (SRMR) absolute fit index was calculated, with values ≤ 0·08 being considered a good fit. Akaike information criterion (AIC) values were also used to compare fit across the models if it was necessary to allow residuals to correlate.

## Results

### Sample characteristics

Overall, 528 children completed the online questionnaire. Sample characteristics are given in Table 1. Children used commercial screen media for an average of 35·0 h per week and non-commercial screen media for 19·5 h per week. Data for 126 participants were removed as they reported implausible media use. Two participants did not report their sex or reported as non-binary and were removed, as these groups were not large enough to generate regression coefficients and would have resulted in separation. This gave a final sample size of 400.

### Main model

#### Hypothesised model 1 (using injunctive norms)

**Fig. 1:**
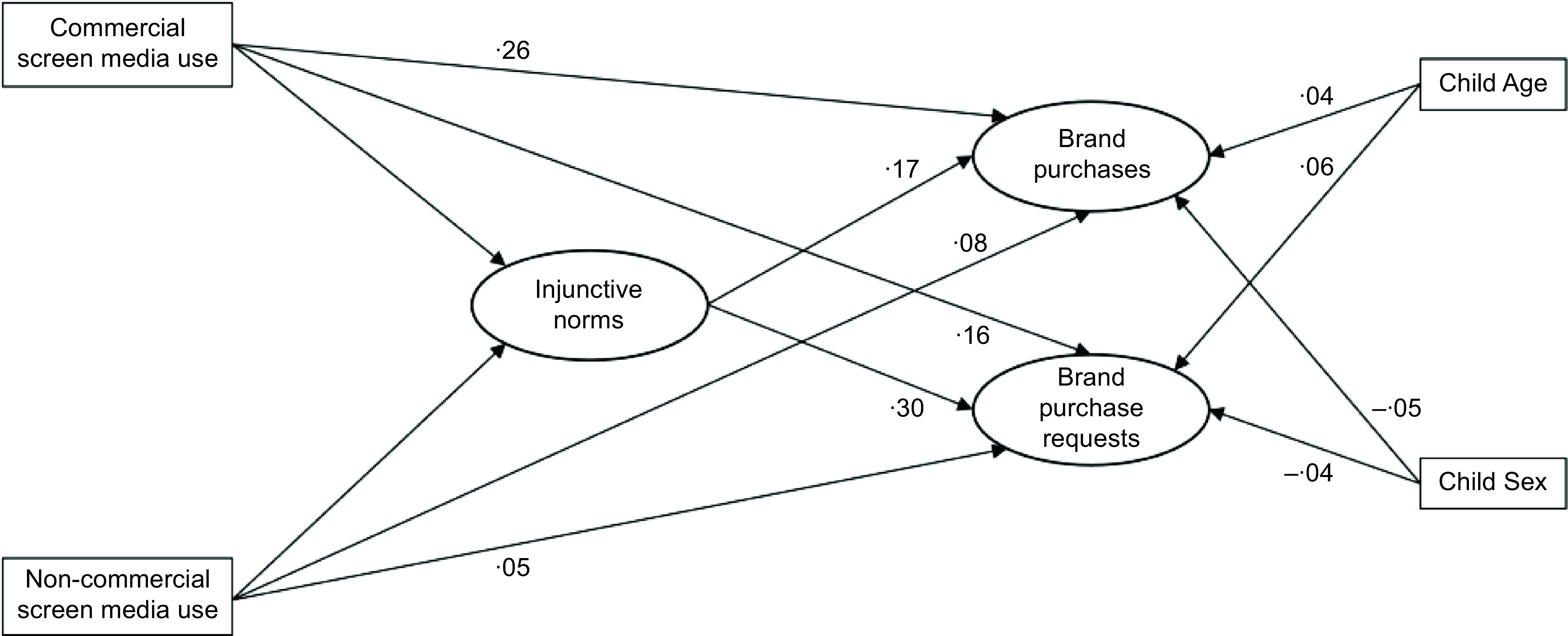
Hypothesised model

The full model (Fig. 1) was a moderate to good fit to the data (Χ^2^(330) = 1005·26, *P* < 0·001, Χ^2^/df = 3·05, CFI = 0·90, RMSEA = 0·072, SRMR = 0·052). We controlled for the covariance between brand purchases and requests in the model (*β* = 0·475, *P* < 0·001). The final model excluded latent variables for brand preferences and brand consumption. Adding these variables to the model led to problematic model fit due to correlated residual confounding. Brand consumption was used in place of brand purchase in an additional model (see Alternative model 1 below).

There was a significant positive correlation between children’s commercial screen media use and their injunctive brand norms, which in turn predicted brand purchasing and requests to parents (Table 2). Commercial screen media use was also directly associated with children’s brand purchasing and requests. Non-commercial screen media use was not associated with injunctive norms about brand, brand purchases nor purchase requests. Descriptive statistics for the outcome variables and factor analyses for constructing the latent variables are given in the online Supplemental Material.

**Table 2:** Direct associations between variables

Association	B	se	*P*	95 % CI
Commercial screen media use > Injunctive norms	0·003	0·001	0·002	0·001, 0·005
Non-commercial screen media use > Injunctive norms	0·002	0·002	0·207	–0·001, 0·005
Commercial screen media use > Brand purchases	0·013	0·003	<0·001	0·007, 0·019
Non-commercial screen media use > Brand purchases	0·006	0·004	0·089	–0·001, 0·013
Injunctive norms > Brand purchases	0·475	0·163	0·003	0·156, 0·794
Child age > Brand purchases	0·030	0·032	0·346	–0·033, 0·094
Child sex > Brand purchases	–0·145	0·132	0·272	–0·404, 0·114
Commercial screen media use > Brand purchase requests	0·008	0·003	0·006	0·002, 0·014
Non-commercial screen media use > Brand purchase requests	0·004	0·004	0·303	–0·004, 0·011
Injunctive norms > Brand purchase requests	0·869	0·172	<0·001	0·533, 1·206
Child age > Brand purchase requests	–0·043	0·035	0·220	–0·113, 0·026
Child sex > Brand purchase requests	–0·128	0·142	0·369	–0·407, 0·151

The indirect effects of commercial screen media use on purchasing (*P* = 0·012) and requests (*P* = 0·002), through the pathway of injunctive norms about brands, were significant.

Skipping ads: To control for children skipping over, muting or blocking advertisements in commercial screen media, we extended the model to include if children used strategies to avoid marketing as an additional predictor of brand attitudes, and brand purchase requests and purchases. Eighteen participants failed to answer this question and were excluded from the analysis. The model was a moderate to good fit (Χ^2^(351) = 1008·49, *P* < 0·001, Χ^2^/df = 2·73, CFI = 0·90, RMSEA = 0·070 SRMR = 0·049). The use of strategies to avoid marketing in commercial screen media was directly associated with reduced injunctive norms about brands (B = –0·062, se = 0·022, *P* = 0·005, 95 % CI –0·106, –0·019). The use of strategies to avoid marketing was also directly associated with lower brand purchases (B = –0·178, se = 0·056, *P* = 0·001, 95 % CI –0·287, –0·069), although commercial screen media use remained associated with brand purchases. The use of strategies to avoiding marketing was not associated with reduced brand purchase requests (B = –0·087, se = 0·057, *P* = 0·130, 95 % CI –0·199, 0·026).

Other brand exposures: Given the different properties of the screen media use and ‘other brand exposure’ variables, we ran separate models to explore the effect of these different brand exposures on the outcomes. That is, the measure of screen use was a proxy for exposure to food marketing generally, while the measure for other brand exposures captured exposure to marketing for specific brands. Further, the measures used different scales, with screen use measured in time (min/d) and other brand exposures using a dichotomous (Y/N) response. This alternative model that explored how other brand exposures influenced injunctive norms and brand purchases and parent purchase requests was an acceptable to good fit (Χ^2^(351) = 1049·95, *P* < 0·001, Χ^2^/df = 2·99, CFI = 0·90, RMSEA = 0·071, SRMR = 0·050). In this model, other brand exposure had a positive association with children’s injunctive norms about brands (B = 0·029, se = 0·010, *P* < 0·001, 95 % CI 0·011, 0·048). Other brand exposure was also associated with increased brand purchase requests (B = 0·126, se = 0·030, *P* < 0·001, 95 % CI 0·067, 0·185) and increased brand purchases (B = 0·152, se = 0·030, *P* < 0·001, 95 % CI 0·093, 0·211).

A separate model was constructed that used children’s descriptive norms about brands (‘Lots of people my age like [brand]’) in place of injunctive norms to represent children’s attitudes to brands. The results of this analysis were identical to model 1, whereby children’s commercial screen media use (but not non-commercial screen media use) had a significant positive association with children’s descriptive norms about brands and children’s brand purchasing and requests (see online Supplemental Material). Descriptive norms about brands were also directly associated with brand purchasing and requests. The indirect effects of commercial screen media use on purchasing and requests, through the pathway of descriptive norms about brands, were again significant. Other brand exposures were also positively associated with children’s descriptive norms about brands, brand purchase requests and brand purchases.

### Alternative models

#### Alternative model 1: Including brand consumption

When consumption was included in the main model together with the brand purchase variable, there were major issues with correlated residuals between every brand on each latent variable (Multiple imputation (MI) > 100). This was likely to be because if children were buying foods with their own money, they were also consuming them^(26)^. There were also problematic correlated residuals with brand purchase requests and brand consumption for the same reasons (lower but still substantial MI, ranging from 90 to 131 across the eight brands, excluding the energy drink brands). Brand consumption was therefore excluded from the main model. An alternative model including brand consumption instead of brand purchasing behaviours was well-fitted (Χ^2^(50) = 103·65, *P* < 0·001, Χ^2^/df = 2·07, CFI = 0·97, RMSEA = 0·057, SRMR = 0·045). In this alternative model that included brand consumption but not brand purchases or purchase requests, both commercial screen media use (B = 0·010, se = 0·002, *P* < 0·001, 95 % CI 0·006, 0·015) and non-commercial screen media use (B = 0·006, se = 0·003, *P* = 0·029, 95 % CI 0·001, 0·012) significantly predicted brand consumption.

#### Alternative model 2: Including other measures of emotional responses to brands

Children’s emotional responses to brands as given using the emoticon scale were used in place of their normative beliefs about brands in a further alternative model. The full model was a moderate to good fit to the data (Χ^2^(329) = 986·99, *P* < 0·001, Χ^2^/df = 3·00, CFI = 0·91, RMSEA = 0·071, SRMR = 0·054). Emotional responses to brands did not predict purchasing behaviours and were not influenced by media exposure.

## Discussion

This study sought to test potential pathways along a theoretical model of effects of children’s food marketing exposure. These pathways spanned proximal effects of marketing, including brand attitudes, to more distal outcomes, including food consumption.

Commercial screen media exposure was strongly positively associated with children’s normative brand beliefs, including whether they perceived that many children their age liked the brands (descriptive norm) and that popular children their age consumed the brands (injunctive norm). Earlier reviews of the evidence on the impacts of food marketing on children have found that marketing influences diet-related social norms, or the foods and food practices that are perceived to be socially normal, agreed and acceptable^(27)^. Commercial screen media use was also directly and indirectly associated with children’s purchases and purchase requests to parents for frequently marketed food and beverage brands, through the pathway of brand beliefs. Children’s self-reported average commercial screen media use was 35 h per week. Given the regression coefficient represents a one-unit change in the independent variable, typical commercial screen use is estimated to be associated with a change food purchases by 0·5 units on the 7-point scale from ‘never’ to ‘every day’. For most brands (except Red Bull and Monster Energy), the mean purchase frequency was between 4·5 and 5·1. Exposure to marketing during usual commercial screen use would be associated with an increase in brand purchases from ‘less often’ to ‘every few weeks’ or ‘every month or so’. Non-commercial screen media use was not associated with children’s brand beliefs, purchases nor requests. In another model that explored the association between other food brand exposures (such as sports sponsorship and outdoor advertising) and brand-related outcomes, other exposures to brands were also strongly associated with children’s brand beliefs and their brand purchases and purchase requests.

Children’s use of strategies to avoid advertising, by skipping over, muting or blocking advertisements, significantly reduced the associations between commercial screen media use and normative beliefs about brands, and between commercial screen media use and brand purchases. However, after controlling for these advertising avoidance strategies, commercial screen media use remained associated with brand purchases and requests. This finding is somewhat expected, given that marketing avoidance is not easy nor possible for all commercial media, especially digital media and on-demand television. Marketers use a range of strategies to counter advertising avoidance, including: preventing users from accessing web content if ad-blockers are detected, providing subscription options, where users have to pay to avoid advertising (e.g. YouTube Premium) and embedding marketing within online content^(28)^. This latter strategy includes embedding marketing in content shared through social media sites, which is then transmitted through online networks and communities (‘earned media’)^(29)^. Australian children have extensive exposure to unhealthful food marketing through earned media online, with each hour of online media use associated with a median of ten exposures to earned media food marketing, mostly promoting unhealthful foods^(23)^. In the current study, online commercial media contributed by far the greatest amount of children’s reported screen use time, for which food marketing would be largely unavoidable. Besides active avoidance of marketing, necessary conditions for children to resist the influence of food marketing include not only an awareness of the persuasive intent of marketing but also the cognitive and emotional ability and motivation to resist marketing^(30)^. The nature of ‘earned media’ marketing in digital media, in which marketing gains further reach by being shared between peers and beyond (e.g. ‘going viral’), blurs the boundary between marketing content and online peer content, obstructing marketing awareness and cognitive, emotional and motivational defences^(31)^.

Our findings build upon other recently published studies on the pathways of effect of food marketing on children^(8–10)^ by assessing the effects of online media and other marketing exposures and by exploring food brand-specific outcomes. Aligned with our findings, British children’s commercial television use was significantly associated with children’s purchase requests, purchases and consumption of unhealthy foods, while non-commercial television was only weakly associated with these outcomes^(8)^. In another study, British children’s recall of unhealthy food marketing on online videogaming livestreaming platforms was significantly associated with their purchase and consumption of frequently marketed food categories, with attitudes towards these unhealthy foods mediating this effect^(10)^. Similarly, in adolescents from the USA, television use was significantly and positively associated with attitudes towards frequently promoted food brands and unhealthy food consumption^(9)^.

In the current study, children’s attitudinal and affective responses to brands were assessed using multiple measures, each having a different bearing on the model outputs. In the final model, children’s normative beliefs about brands were significantly associated with marketing exposure (commercial screen media use and other brand exposures) and with other more distal outcomes (brand purchases and requests). In an earlier study with adolescents in the USA, in which there was a positive association between attitudes towards frequently promoted food brands and unhealthy food consumption, brand attitudes were measured using sematic differential scales to describe what children thought about each brand (e.g. very cool to very uncool) and injunctive norms (the popularity of the type of person who would consume the brand)^(9)^. In an alternative model in the current study, children’s emotional response to the brands, as reported using an emoticon scale, was not associated with children’s food brand purchases or purchase requests, nor with children’s marketing exposures. Emoticons have been found in earlier studies to predict food product selection from a choice set^(32)^ and to be a familiar measure of food-related emotions for pre-adolescent and adolescent children^(17)^. However, despite this and the extensive formative testing of the questionnaire, the different associations between variables measuring brand attitudes and affective responses to brands highlight the challenge of accurately capturing emotional responses to brands, especially using quantitative measures, in a way that is meaningful for children. It also emphasises the unconscious nature of implicit brand affect, which may be better detected through other non-survey methods^(33)^.

A number of studies have assessed the effect of children’s food marketing exposure on food preferences, with meta-analyses identifying a significant but small effect^(4)^. Measures of food preference have included the selection of food from a choice set and indicators of liking of a food on Likert or semantic differential scales. In the current study, we originally included a measure of children’s food preferences, in which we asked children if they would serve the reference brands at a party and if they would be willing to pay more for the branded foods compared with similar but unbranded foods. However, the inclusion of this variable in the model led to problematic residuals, likely as if children had positive attitudes towards the brand they also preferred it.

Accurate measures of children’s screen media use are necessary for estimating children’s exposure to food marketing on these media. Children in our study reported higher screen use behaviours compared with other Australian studies. In a survey of almost 4000 Australian children aged up to 18 years, average weekly screen use was 31·5 h for 6–12 year olds and 43·6 h for 13–18 year olds^(34)^. This compares to 54·6 h per week across commercial and non-commercial screen media in the current study. Higher screen media use was likely related to COVID lockdowns at the time of the study^(35)^. Notably, in the other survey, parents reported children’s screen use by proxy, which may have led to an underestimation, particularly for older children. Survey measures that ask children to recall their media use on a ‘typical’ day are frequently used to assess media use behaviours, although they have been found to overestimate actual media use^(36)^. Screen multi-tasking, whereby children simultaneously access multiple platforms across devices, is common^(37)^ and further complicates children’s recall of media use. Future studies exploring the effects of screen-based food marketing exposure should consider other, potentially more valid, measures of screen use behaviours, including media diaries^(38)^.

The study had a number of limitations. The use of purposive sampling from the research survey panel was potentially less ideal for obtaining a representative sample that other random sampling approaches. However, other sampling methods were less feasible for achieving the large sample size. We also set quotas for child age, sex and geographic location to improve the diversity of participants as aligned with population characteristics. The cross-sectional study design precludes causal inferences between children’s marketing exposure and diet-related outcomes. Future research should prioritise the conduct of well-controlled longitudinal studies that can establish temporality between exposure and outcomes and account for residual confounding between the outcomes of interest. Our analyses were limited by poor fit of the data when variables related to food brand preferences, brand attachment and brand consumption were included. Poor fit was a result of variance that could not be explained by the variables in the model. Children’s dietary behaviours are influenced by a complex array of factors, including their socio-economic status, parental feeding practices and mother’s weight status among others^(39)^, some of which may be controlled for in longitudinal studies. Of the ten reference brands used in the survey, the two energy drink brands exhibited a different pattern of responses for brand purchases and purchase requests and were thus excluded from the models. Other surveys have found that only 8 % of Australian adolescents report consuming energy drinks at least weekly^(40)^. Energy drink consumption has been associated with poorer sleep behaviours in adolescents^(40)^ and digital marketing of energy drinks is linked to energy drink use^(41)^. However, given the relative infrequency of consumption (and therefore purchase) of these drinks in this age group, alternative beverage brands would have been more appropriate to test in the current survey.

Children’s exposure to marketing on commercial screen media, including television and online platforms, and other food brand exposures, including through sponsorships and outdoor advertising, were associated with their brand beliefs and purchasing behaviours of frequently promoted unhealthful foods and beverages. Purposeful avoidance of advertising in screen media reduced but did not remove the association between exposure to marketing on these outcomes. Children’s avoidance of marketing is not practical nor possible for most screen media content. Regulations that restrict children’s exposures to unhealthy food marketing, both for on-screen media and through other media, are required to reduce the effect of marketing exposure on children’s food purchasing behaviours.

In this study, testing of some of the outcomes along the Hierarchy of Effects model of food marketing was impaired by residual confounding, including for brand preferences and brand consumption, which led to problematic model fit. Well-designed longitudinal studies are needed, which can test the outcomes of interest while controlling for other factors associated with marketing exposure and responses to food brands. Nevertheless, the findings of this study support the relationships between children’s food marketing exposure and food brand-related outcomes along the Hierarchy of Effects pathways. Specifically, exposure to marketing for food and beverage brands on-screen and through other media was associated with children’s perceptions that these brands were normal and socially desirable, and this was associated with their purchase of these brands and purchase requests to parents.

## Supporting information

Kelly et al. supplementary material 1 Kelly et al. supplementary material

Kelly et al. supplementary material 2 Kelly et al. supplementary material

## Supplementary material

For supplementary material accompanying this paper visit https://doi.org/10.1017/S1368980023002616
