# Supplementary figures and images for: Testing a conceptual Hierarchy of Effects model of food marketing exposure and associations with children and adolescents’ diet-related outcomes
Source: Public Health Nutr. 2023 Dec 7;27(1):e10. doi: 10.1017/S1368980023002616 (PMC10830363; doi:10.1017/S1368980023002616)

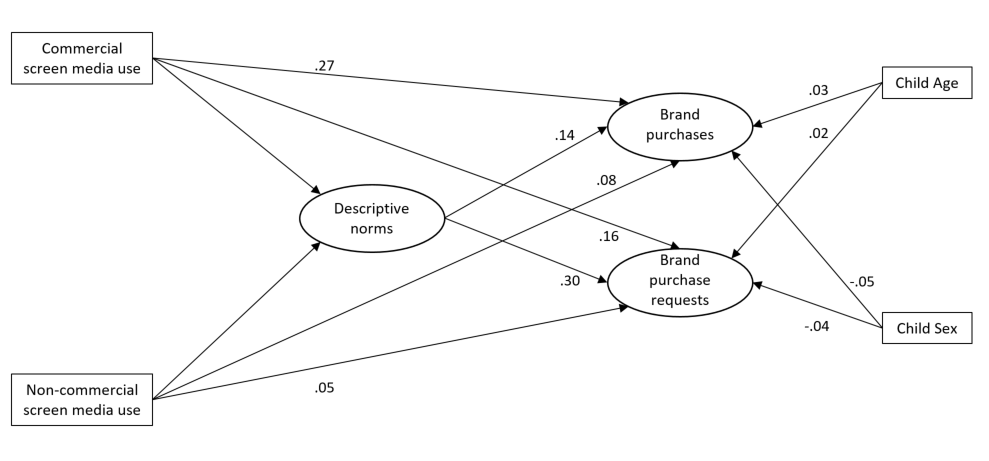


Figure S1: Model using descriptive norms

Supplement: Kelly et al. supplementary material 1 — Kelly et al. supplementary material [file S1368980023002616sup001.docx]
