# Supplementary material for: Testing a conceptual Hierarchy of Effects model of food marketing exposure and associations with children and adolescents’ diet-related outcomes
Source: Public Health Nutr. 2023 Dec 7;27(1):e10. doi: 10.1017/S1368980023002616 (PMC10830363; doi:10.1017/S1368980023002616)
Supplement: Kelly et al. supplementary material 2 — Kelly et al. supplementary material [file S1368980023002616sup002.docx]

**Supplementary material**

*Descriptive statistics for the outcome variables*

Table S1: Mean (SD) responses to brand-related outcomes, by food and drink brands

| Variable | Mean ±SD | | | | | | | | | |
| --- | --- | --- | --- | --- | --- | --- | --- | --- | --- | --- |
|  | McDonalds | KFC | Pringles | Twisties | Kit Kat | M&Ms | Maltesers | Coca Cola | Red Bull | Monster Energy |
| Emotional responses to the brands |  |  |  |  |  |  |  |  |  |  |
| Injunctive norms ^a^ | 4.13(1.05) | 3.99(0.99) | 3.66(1.03) | 3.63(1.05) | 3.82(1.02) | 3.85(0.98) | 3.69(1.06) | 3.86(1.11) | 2.63(1.40) | 2.60(1.37) |
| Descriptive norms ^a^ | 4.28(0.97) | 4.06(1.03) | 3.84(1.01) | 3.75(1.02) | 3.95(0.99) | 4.00(0.95) | 3.82(1.06) | 3.99(1.09) | 2.66(1.41) | 2.66(1.40) |
| Emoticon ^b^ | 5.05(1.75) | 5.05(1.71) | 4.86(1.53) | 4.55(1.54) | 5.05(1.53) | 4.99(1.54) | 4.94(1.59) | 4.59(1.80) | 3.11(1.60) | 3.02(1.60) |
| Brand purchase requests ^c^ | 2.83(1.89) | 2.62(1.74) | 2.36(1.66) | 2.27(1.64) | 2.38(1.66) | 2.37(1.65) | 2.33(1.66) | 2.42(1.81) | 1.52(1.28) | 1.59(1.39) |
| Brand purchases ^c^ | 2.12(1.68) | 1.93(1.55) | 1.84(1.45) | 1.83(1.46) | 1.96(1.54) | 1.93(1.51) | 1.88(1.46) | 2.06(1.71) | 1.51(1.36) | 1.59(1.39) |
| Brand consumption ^c^ | 3.82(1.57) | 3.47(1.55) | 3.30(1.49) | 3.26(1.52) | 3.15(1.51) | 3.40(1.45) | 3.31(1.50) | 3.70(1.83) | 2.05(1.75) | 2.07(1.72) |

Acronyms: SD, standard deviation

^a^ 5-point scale from ‘strongly disagree’ to ‘strongly agree’; ^b^ 7-point scale from ‘hate’ to ‘love’; ^c^ 7-point scale from ‘never’ to ‘every day’

*Constructing latent variables*

Emotional responses to brands

Inclusion of all dimensions of emotional responses to brands from the questionnaire led to poor fit of the model. As such, in the final model emotional responses to brands was limited to children’s normative beliefs about brands.

Injunctive norms: The initial confirmatory factor analysis was a poor fit, (Χ^2^(20) =107.31, p<.001, Χ^2^/df= 5.37, CFI =.88, RMSEA = .10, SRMR = .074; AIC=8325.98). Inspection of modification indices suggested that the residuals between McDonalds and KFC (MI=68.09) and McDonalds and Coca cola (MI=35.91) were correlated. We ran the analysis including covariance’s between these residuals. This gave an improved fit (Χ^2^(18) =44.58, p<.001, Χ^2^/df= 2.48, CFI =.96, RMSEA = .06, SRMR = .048; AIC=8233.67) with all items having a highly significant positive loading on the latent variable (β’s>.482, p’s<.001). Scale ω_T_= .89.

Descriptive norms: The initial confirmatory factor analysis was a relatively poor fit, (Χ^2^(20) =93.42, p<.001, Χ^2^/df= 4.67, CFI=.89, RMSEA = .10, SRMR = .066; AIC=8318.70). Inspection of modification indices suggested that the residuals between McDonalds and KFC (MI=58.84) and McDonalds and Coca cola (MI=37.17) were correlated. We ran the analysis including covariances between these residuals. This gave an improved fit (Χ^2^(18) =29.54, p<.001, Χ^2^/df= 1.64, CFI =.98, RMSEA = .04, SRMR = .039; AIC=8228.54) with all items having a highly significant positive loading on the latent variable (β’s>.447, p’s<.001). Scale ω_T_= 0.87.

Brand purchase requests

The initial confirmatory factor analysis for parent purchase requests showed a largely poor fit to the data (Χ^2^(35) =181.66, p< 0.001, Χ^2^/df= 5.19, CFI = 0.90, RMSEA = 0.103, SRMR = 0.064; AIC = 11885.13). Inspection of modification indices suggested that this was largely due to correlated residuals for Red Bull and Monster Energy drinks (MI=231.40). Furthermore, responses to these brands were substantially different to all other products, with ‘never’ being the median and upper hinge response. We therefore removed these two brands from the analyses. The fit was substantially improved (Χ^2^(20) =61.36, p<0.001, Χ^2^/df=3.07, CFI = 0.96, RMSEA = .072, SRMR = .033; AIC=9637.13). Based on modification indices (MI=75.19) we allowed the residuals between McDonalds and KFC to correlate, giving an excellent final fit (Χ^2^(19) =34.35, p=.017, Χ^2^/df= 1.81, CFI =.99, RMSEA = .045, SRMR = .024; AIC = 9561.99) with all items having a highly significant positive loading on the latent variable (β’s>0.770, p’s<0.001). Scale ω_T_= .97.

Brand purchases

The initial confirmatory factor analysis for children’s own purchases showed a moderate fit to the data (Χ^2^(35) =111.83, p<0.001, Χ^2^/df= 3.20, CFI =.95, RMSEA = .074, SRMR = .036; AIC = 10164.42). In an identical pattern as the previous analysis, the two energy drinks were again problematic (in terms of MI=126.99 and distribution) and were removed from analyses. The fit was substantially improved (Χ^2^(20) =56.74, p<.001, Χ^2^/df= 2.84, CFI =.97, RMSEA = .068, SRMR = .030; AIC= 8181.18), although modification indices (MI=57.26) again suggested that the residuals between McDonald’s and KFC should be correlated, producing a final model that was an excellent fit to the data (Χ^2^(19) =38.17, p=.016, Χ^2^/df=2.01, CFI =.98, RMSEA = .050, SRMR = .027; AIC= 8125.06), with all items showing a highly significant loading on the latent variable (β’s>.797, p<.001). Scale ω_T_= .98.

*Hypothesised Model 2 (using descriptive norms)*

The full model (Figure S1) was a moderate to good fit to the data (Χ^2^(330) =941.34, p<0.001, Χ^2^/df=2.85, CFI =.91, RMSEA = .068, SRMR = .047). We controlled for the covariance between brand purchases and requests in the model (β =0.481, p<.001). Identical to Model 1 in the manuscript, there was a significant positive correlation between children’s commercial screen media use and their descriptive norms about brands, which also predicted purchasing and requests (Table S1). Again, commercial screen media use was directly associated with children’s brand purchasing and requests. Non-commercial screen media use was not associated with descriptive norms about brands, brand purchases nor purchase requests.

Table S2: Direct associations between variables

| Association | B | SE | p | 95% CI |
| --- | --- | --- | --- | --- |
| **Commercial screen media use > Descriptive norms** | **.003** | **.001** | **.002** | **.001 to .006** |
| Non-commercial screen media use > Descriptive norms | .002 | .002 | .248 | -.001 to .005 |
| **Commercial screen media use > Brand purchases** | **.013** | **.003** | **<.001** | **.007 to .019** |
| Non-commercial screen media use > Brand purchases | .006 | .004 | .085 | -.001 to .013 |
| **Descriptive norms > Brand purchases** | **.397** | **.153** | **.010** | **.097 to .679** |
| Child age > Brand purchases | .022 | .032 | .498 | -.041 to .085 |
| Child sex > Brand purchases | -.137 | .133 | .302 | -.398 to .123 |
| **Commercial screen media use > Brand purchase requests** | **.008** | **.003** | **.005** | **.002 to .014** |
| Non-commercial screen media use > Brand purchase requests | .004 | .004 | .311 | -.004 to .012 |
| **Descriptive norms > Brand purchase requests** | **.868** | **.184** | **<.001** | **.508 to 1.229** |
| Child age > Brand purchase requests | -.053 | .035 | .116 | -.124 to .014 |
| Child sex > Brand purchase requests | -.112 | .143 | .432 | -.392 to .167 |

Acronyms: SE, standard error; CI, confidence interval

The indirect effects of commercial screen media use on purchasing (p=.027) and requests (p=.003), through the pathway of descriptive norms about brands, were significant.

Insert Figure S1 here

Skipping ads: Again, we extended the model to control for children skipping over, muting or blocking advertisements in commercial screen media. The model was a moderate to good fit (Χ^2^(351) =926.50, p<.001, Χ^2^/df=2.73, CFI =.92, RMSEA = .066 SRMR = .045). The use of strategies to avoid marketing in commercial screen media was directly associated with lower brand purchases (B=-0.188, SE=0.056, p=.001, 95%CI = -0.297 to -0.079), although commercial screen media use remained associated with brand purchases. The use of strategies to avoid marketing was not associated with reduced descriptive norms (B=-0.043 SE=0.022, p=.071, 95%CI = -0.213 to 0.009) nor brand purchase requests (B=-0.102, SE=0.057, p=.071, 95%CI = -0.213 to 0.009).

Other brand exposures: We again ran a separate model that included other brand exposures rather than screen media use. This model was an acceptable to good fit (Χ^2^(351) =979.48, p<.001, Χ^2^/df= 2.79, CFI =.91, RMSEA = .067, SRMR = .046). As with Model 1 in the manuscript, in this model other brand exposure had a positive association with children’s descriptive norms about brands (B=.032, SE=.010, p=.001, 95%CI = .013 to .052). Again, other brand exposure was also associated with increased brand purchase requests (B=.123, SE=.030, p<.001, 95%CI = .064 to .1825) and increased brand purchases (B=.153, SE=.030, p<.001, 95%CI = .064 to .182).
